# Total daily energy expenditure and elevated water turnover in a small-scale semi-nomadic pastoralist society from Northern Kenya

**DOI:** 10.1080/03014460.2024.2310724

**Published:** 2024-03-04

**Authors:** Amanda McGrosky, Zane S. Swanson, Rebecca Rimbach, Hilary Bethancourt, Emmanuel Ndiema, Rosemary Nzunza, David R. Braun, Asher Y. Rosinger, Herman Pontzer

**Affiliations:** a Evolutionary Anthropology, Duke University, Durham, NC, USA; b Global Food and Water Security Program, Center for Strategic and International Studies, Washington, WA, USA; c Department of Behavioural Biology, University of Münster, Münster, Germany; d Department of Anthropology, Northwestern University, Evanston, IL, USA; e Department of Earth Sciences, National Museums of Kenya, Nairobi, Kenya; f Kenya Medical Research Institute, Nairobi, Kenya; g Center for the Advanced Study of Human Paleobiology, Anthropology Department, George Washington University, Washington, WA, USA; h Technological Primate Research Group, Max Planck Institute for Evolutionary Anthropology, Leipzig, Germany; i Department of Biobehavioral Health, PA State University, University Park, PA, USA; j Department of Anthropology, Pennsylvania State University, State College, PA, USA; k Duke Global Health Institute, Duke University, Durham, NC, USA

**Keywords:** Energy expenditure, doubly labelled water, water turnover, pastoralism

## Abstract

**Background:**

Pastoralists live in challenging environments, which may be accompanied by unique activity, energy, and water requirements.

**Aim:**

Few studies have examined whether the demands of pastoralism contribute to differences in total energy expenditure (TEE) and water turnover (WT) compared to other lifestyles.

**Subjects and methods:**

Accelerometer-derived physical activity, doubly labelled water-derived TEE and WT, and anthropometric data were collected for 34 semi-nomadic Daasanach adults from three northern Kenyan communities with different levels of pastoralist activity. Daasanach TEEs and WTs were compared to those of other small-scale and industrialised populations.

**Results:**

When modelled as a function of fat-free-mass, fat-mass, age, and sex, TEE did not differ between Daasanach communities. Daasanach TEE (1564–4172 kcal/day) was not significantly correlated with activity and 91% of TEEs were within the range expected for individuals from comparison populations. Mean WT did not differ between Daasanach communities; Daasanach absolute (7.54 litres/day men; 7.46 litres/day women), mass-adjusted, and TEE-adjusted WT was higher than most populations worldwide.

**Conclusions:**

The similar mass-adjusted TEE of Daasanach and industrialised populations supports the hypothesis that habitual TEE is constrained, with physically demanding lifestyles necessitating trade-offs in energy allocation. Elevated WT in the absence of elevated TEE likely reflects a demanding active lifestyle in a hot, arid climate.

## 1. Introduction

Total daily energy expenditure (TEE; kcal/day) is the sum of all energy expended on physiological tasks. Despite major differences in lifestyle and habitual physical activity levels, fat-free body mass-adjusted TEE is remarkably consistent across modern human populations (Luke et al. 2009; Dugas et al. 2011; Pontzer et al. 2012; 2018; Urlacher et al. 2021). Hadza hunter-gatherers in Tanzania and Tsimane’ forager-farmers in lowland Bolivia, for example, have mass-adjusted TEEs that are indistinguishable from those of more sedentary Western populations (Pontzer et al. 2012; 2018). The elevated TEEs of some populations that deviate from the trend, such as Shuar forager-horticulturalists of the Ecuadorean Amazon (e.g. Christopher et al. 2019), are hypothesised to be linked to cultural practices or high physical activity levels during the measurement period. Across populations and over longer timescales, habitual physical activity levels do not appear to be a strong predictor of lean mass-adjusted habitual TEE (Pontzer 2018). Investigations of TEE using doubly labelled water reveal that TEE is not the simple sum of resting energy expenditure and energy expended in physical activity. Rather, analyses of large adult samples indicate that although physical activity levels and TEE are positively correlated over the short term (i.e. weeks), TEE plateaus over longer time periods (i.e. months) (Pontzer et al. 2016; Pontzer 2018). This suggests that there is a metabolic ceiling to total energy expenditure (Pontzer 2018).

Unlike habitual energy expenditure, which appears to be constrained, daily water requirements and water throughput can vary substantially between populations, particularly across climates and lifestyles (Sawka et al. 2005; Rosinger 2020; Swanson and Pontzer 2020; Yamada et al. 2022). Water is among the most important nutrients required to sustain life as the body uses water to transport nutrients and waste, structure tissues, protect against infection and disease, and regulate temperature (Michaud et al. 1999; Tang et al. 1999; Pearle 2001; Raman et al. 2004). Though hydration is crucial for thermoregulation, adequate hydration can be difficult to maintain in hot, dry environments, where daily water needs may surpass 8 litres/day for active individuals (Sawka et al. 2005). When a hot, dry climate is coupled with scarcity of clean drinking water or other dimensions of water insecurity, challenges to maintaining hydration can compound and necessitate culturally specific hydration strategies (Rosinger 2023). Acute severe water deficits can result in life-threatening dehydration, reduced sweating, increased core body temperature, increased heart rate, and/or heat exhaustion (Noakes 1998; Kleiner 1999). Failure to meet adequate water needs and chronic dehydration have embodied health consequences including oxidative stress, immune function dysregulation, and kidney damage (Roncal-Jimenez et al. 2015).

Pastoralism is an adaptive strategy to living in marginal environments characterised by intentional and regular moves in search of pasture and water (Tugjamba et al. 2023), but few studies have investigated the effect of a pastoralist lifestyle on TEE and daily water turnover (the volume, litres, of body water replaced in one day). This project therefore investigates energy expenditure and water requirements among Daasanach, a semi-nomadic pastoralist group living in southwestern Ethiopia and northwestern Kenya.

The primary objective of this study is to understand both the daily energetic and water requirements of a pastoralist population living a demanding lifestyle in an extreme environment. First, we test whether activity levels, energy expenditure, and water requirements differ between communities with different levels of pastoralist activity. We also explore how Daasanach TEE and water turnover rates compare to those of other small-scale populations practicing different livelihood strategies and those of a larger, worldwide sample. A constrained energy expenditure model (Pontzer 2018) predicts consistent body mass-, age-, and sex-adjusted habitual energy expenditure, regardless of pastoralist activity level and lifestyle. Higher water turnover is often linked to higher levels of physical activity and higher ambient temperatures (Sawka et al. 2005; Yamada et al. 2022), which would predict higher water turnover among Daasanach living in more traditionally pastoralist communities as well as higher water turnover across all Daasanach populations compared to that of populations living in more temperate climates.

## 2. Field site and methods

### 2.1. Daasanach pastoralists

About 48,000 Daasanach live in Ethiopia and about 19,000 in Kenya (Mwamidi et al. 2018), with many members of the group in Kenya living in the vicinity of the town of Illeret in the Turkana Basin. The Turkana Basin has been one of the hottest places on earth for at least 3 million years and currently is in the top 1% hottest land areas in the world with a mean annual temperature of 29.2 °C (Passey et al. 2010). In recent years, drought, flash flooding, and climate variability have been increasing, leading to an increased threat of food and water insecurity that may impact the community’s pastoralist movement and practices.

Though Daasanach are described ethnographically as agro-pastoralists, Daasanach in Kenya have few opportunities to practice agriculture and largely rely on maize, beans, and sorghum that is purchased from markets and supplemented with dairy and occasional meat products from goat, cattle, and camel herds (Bethancourt et al. 2022). Traditional Daasanach lifestyles are physically demanding, with men tending to livestock and women bearing responsibility for water collection, cooking, childcare, and home maintenance (Swanson 2021). Lifestyle varies to some degree between Daasanach communities. Communities located close to the main town of Illeret are more sedentary and market-integrated, while those living in the fora (remote satellite grazing camps) practice a more traditional pastoralist lifestyle that may require higher levels of physical activity as communities and households travel with their herds. Particularly for those living in the fora, obtaining water can be particularly time- and labour-intensive, as it is often collected from hand-dug wells in dry riverbeds (lagas) that can be up to 3 or 4 kilometres away (Bethancourt et al. 2022). Fora communities typically drink more milk than more market-integrated communities, and many Daasanach communities have a daily tradition of drinking coffee and/or tea (Sagawa 2006; Rosinger et al. 2021).

### 2.2. Ethical approval and data collection

Ethical approval was obtained from The Pennsylvania State University Institutional Review Board (STUDY00009589), the Kenya Medical Research Institute (KEMRI/RES/7/3/1), the Director of Health in the county government of Marsabit, Kenya, and Daasanach community leaders. All participants provided informed oral and written consent with the help of Daasanach language translators.

Adults were recruited to participate in the summers of 2019 and 2022 (Table 1) from Illeret, Kenya and neighbouring communities. Illeret (4.314°N,36.227°E) is located in Marsabit county on the arid/semi-arid northeastern shore of Lake Turkana. Illeret town and the surrounding region experience bimodal precipitation seasonality, with yearly rainfall averaging about 217 mm and temperatures ranging from 20 °C to 37 °C (Opiyo 2013).

Eligible participants were men and women 18 years or older and residing in the communities of Illeret town (the region’s market centre, where residents are most market integrated), El Bokoch (a community approximately 5 km from the Illeret market centre, where residents have a more traditional lifestyle but have access to a school and can reach Illeret easily), and Roto (a remote fora community approximately 17 km from the Illeret market centre, where residents practice high levels of pastoralist activity and have lower levels of market integration).

Height (measured *via* Seca standing stadiometer), body mass (measured *via* Tanita digital bioimpedance scale) and age (determined using a government ID or through estimation relative to major community events) were recorded for all 34 participants. 12 participants lived in El Bokoch, 12 in Illeret town, and 10 in Roto.

Total energy expenditure (TEE) was measured using doubly labelled water (DLW) following established field sample collection protocols (e.g. Christopher et al. 2019). Each participant provided a baseline urine sample between 1 and 2 h prior to consuming an approximately 80 g dose of DLW (6% ^2^H_2_O, 10% H_2_^18^O) difficulty arranging transportation to remote collection sites or a participant travelling to the fora with their herds). All urine samples were stored in a field freezer before transfer to a −80°C freezer for storage prior to thawing and 30kDalton centrifuge filtering.

Samples were analysed *via* laser absorption mass spectrometry (ABB ICOS) for ^2^H and ^18^O enrichment. All samples were run at least three times; average isotope enrichments were used for subsequent calculations. Water turnover (litres/day) was calculated from ^2^H dilution space (ND) and elimination rate (kD) as $0.01802\times 1.043\times kD\times \frac{ND}{0.99}$ assuming 0.01802 litres/mol H_2_O fractionation correction of 0.99 (Speakman et al. 2021).^2^H and ^18^O depletion rates (kD and kO, respectively) generated using the slope-intercept method were used to calculate CO_2_ production rate (rCO_2_; litres/day) over the measurement period following Speakman et al. (2021):

$${\text{rCO}}_{2}=\left({\text{N}}_{\text{corr}}/2.078\right)*(1.007\text{kO}-1.043\text{kD})-\left(0.0246{\;\text{N}}_{\text{corr}}*\right.\left(1.05\left(1.007\text{k}O-1.043\text{kD}\right)\right),$$

where corrected N $N_{corr}=\frac{\frac{NO}{1.007}+\frac{ND}{1.043}}{2}$ and NO is the ^18^O dilution space (Speakman et al. 2021). rCO_2_ was used to calculate TEE (kcal/day), with $TEE=22.26\times rCO_{2}\times \left(1.106\times \frac{3.94}{FQ}\right)$ (Speakman et al. 2021) assuming a food quotient of 0.86, following previous studies (IAEA. 2009). We note that a food quotient of 0.86 , which is typically used for populations that lack detailed dietary assessments, assumes fats account for ~30% of the daily energy intake (IAEA. 2009).

Total body water (TBW) was determined from isotope dilution $TBW=N_{corr}\times 0.01802$ and was used to calculate fat-free mass (FFM) assuming a hydration constant of 0.732 $FFM=\frac{TBW}{0.732}$. Fat mass (FM) was calculated by subtracting FFM from body mass.

A subsample of DLW participants were asked if they would like to participate in accelerometry measurements using semi-random sampling to ensure a relatively equal distribution of sex and age among accelerometry participants. Five participants from Illeret town (3 women, 2 men) and eight participants from the Roto community (3 women, 5 men) were equipped with ActiGraph GT3X + accelerometers operating at a 30 Hz sampling rate (10 s epoch) to quantify physical activity level and steps per day during the DLW measurement period. Participants were instructed to wear accelerometers for the full week of data collection, including while sleeping. Due to the timing of accelerometer deployment and the number of accelerometers available, it was not feasible to deploy accelerometers in El Bokoch.

### 2.3. Analytical methods

All analyses were performed in R version 4.2.1. TEE, FFM, and FM were natural log-transformed prior to all TEE analyses. InTEE was modelled as a function of InFFM, InFM, age, sex, and/or community in multiple linear regression models using R’s *Im* function. The residuals of the best-fitting multiple linear regression model as identified using adjusted R^2^ (lnTEE ~ lnFFM + lnFM + sex) were also used to represent body mass- and sex-adjusted TEE in an ANOVA to evaluate community-level differences in TEE.

To understand how Daasanach TEE compared to TEE in other active small-scale societies from warm to hot climates, we used published data collected from Hadza (Pontzer et al. 2015), Shuar (Christopher et al. 2019), and Tsimane’ (Gurven et al. 2016) communities. To test for differences in TEE between populations, population was included as a predictor in a multiple linear regression model of InTEE as a function of InFFM, InFM, age, and sex. Each Daasanach participant’s TEE was also predicted from their FM, FFM, age, and sex using Pontzer et al. (2021)’s models. These models were generated from a large, comprehensive dataset of over 6000 sedentary and active adults aged 20–60years across modern human populations. To test whether Daasanach TEEs were significantly higher or lower than would be expected relative to this worldwide sample, R’s *predict* function was used to calculate point estimates and 95% prediction intervals from the Pontzer et al. (2021) models at Daasanach participant’s FM, FFM, age, and sex.

Daasanach activity data were converted to mean steps per day (steps) and minutes of moderate to vigorous physical activity per day (MVPA) using the *PhysicalActivity* package (Choi et al. 2021). Accelerometry data were first cropped to the start and end times of the 7-day DLW measurement period and further cropped to wear time using the *wearingMarking* function and classified light or moderate-vigorous physical activity using the vector magnitude cut points of Sasaki et al. (2011; Keadle et al. 2014). Moderate-vigorous physical activity (MVPA) was identified by a vector magnitude ≥2690, light physical activity identified by a vector magnitude ≥200 and <2689, and sedentary activity was identified by a vector magnitude <200. We expect that some non-wear minutes are also included in this <200 vector magnitude sedentary minutes metric, so we hereafter refer to it as sedentary+nonwear. Because some participants did not wear their accelerometer for the full 7 days (88.5% compliance across 70 total wear days), sedentary+nonwear minutes, light minutes and MVPA minutes are reported as mean daily values averaged over the measurement period (see Supplementary Table S1 for full accelerometry data). While including InFFM, InFM, sex and/or age in multiple linear regressions, InTEE was modelled as a function of (a) steps and (b) daily mean MVPA minutes to test for activity-linked differences in energy expenditure. Differences in mean steps per day between Daasanach communities (Illeretand Roto) were evaluated using a non-parametric Kruskal-Wallis test due to small sample size.

Daily water turnover was modelled as a function of FFM, FM, age, and/or sex, as well as a function of TEE and mean minutes MVPA. Models were run both with and without community (Roto, El Bokoch, Illeret) as a predictor. Population-level differences in water turnover were assessed using multiple linear regression models of turnover as a function of TEE, population, sex, and/or population. Population- and sex-linked differences in mean water turnover were also evaluated using residuals of a turnover TEE linear model to represent TEE-adjusted turnover in an ANOVA of adjusted water turnover across populations by sex (e.g. Daasanach men, Daasanach women, Shuar men, Shuar women, etc.). See Table S2 for details of comparison populations.

To further explore between-community and between-population differences in TEE and water turnover, TEE and water turnover models were repeated with community (Illeret, El Bokoch, Roto) or population (Daasanach, Hadza, Shuar, Tsimane) as an interaction term in the supplementary information (Tables S3, S4).

## 3. Results

Of the 34 participants, 7 women and 5 men lived in Illeret town, 6 women and 6 men lived in El Bokoch, and 6 women and 4 men lived in Roto. Each participant’s age (range: 20–56 years; mean: 32 years), sex (19 women, 15 men), body mass (mean: 52.5 kg men, 49.2 kg women), body composition (mean: 14% body fat men, 25% body fat women), TEE (mean: 2630 kcal/day men, 2325 kcal/day women), and water turnover (mean: 7.36 litres/day men, 7.26 litres/day women) are presented in Table 1.

### 3.1. TEE

**Figure 1.**
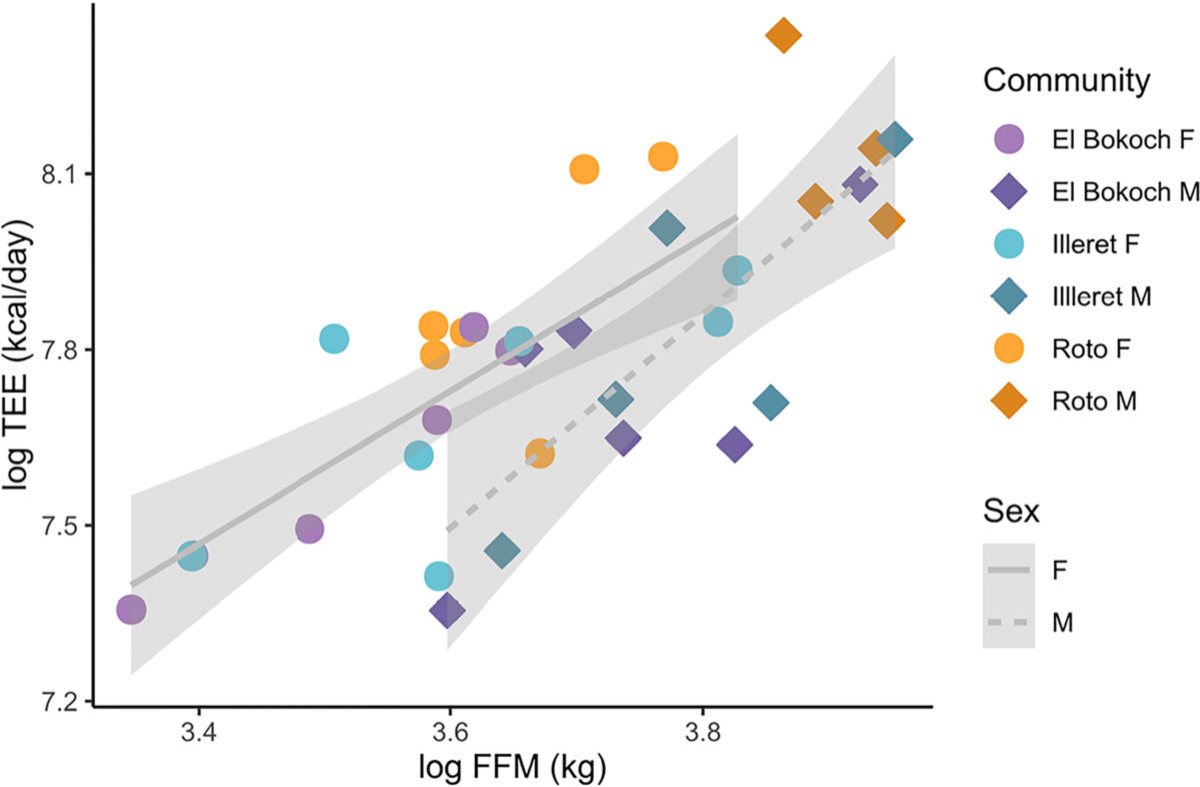
Daasanach TEE (kcal/day) increases with FFM (kg) (R^2^ = 0.54; *p* < 0.001). There are no significant differences in absolute or FFM-adjusted TEE between communities, but Daasanach women (circles) have higher TEE for lean mass than Daasanach men (diamonds).

Among Daasanach participants, TEE was positively correlated with FFM, with women having a higher lean mass-adjusted TEE than men (Figure 1; Table 2). Daasanach TEE was best modelled as a function of FFM, FM, and sex (adjusted R^2^ = 0.65; Table 2). When age was added to the best-fitting model, model fit (i.e. adjusted R^2^) decreased (Table 2). When testing community-level differences in TEE, community was not a significant predictor of TEE (Table 2) and there were no statistically significant differences in body mass- and sex-adjusted TEE between the Illeret, El Bokoch, and Roto communities (ANOVA F-stat = 0.521, df = 2).

In multiple regression models of TEE across small-scale societies, the only significant non-population predictor of TEE was FFM; relative to Daasanach participants, only being a member of the Hadza population had a significant effect on TEE (Table 3). When individual Daasanach TEEs are predicted from participants’ FFM, FM, age, and sex using Pontzer et al. (2021) models generated from a global sample, 29 of 32 observed TEEs fall within the predicted TEE range (Figure 3). The observed TEEs of 3 individuals (1 man, 2 women), all from the Roto community, were higher than the TEE predicted from Pontzer et al. (2021) (Figure 3).

Mean steps per day ranged from 3059 to 15,776 steps (Table 1), with men taking on average more steps than women in both the Roto and Illeret communities (Roto men mean = 8222 steps; Illeret men mean = 13,310 steps; Roto women mean = 4585 steps; illeret women mean = 5608 steps). Differences in mean steps across communities by sex, however, were not statistically significant (Kruskal-Wallis X^2^= 6.25; df = 2). In multiple regression models that included FFM, FM, age, and/or sex, neither mean steps per day nor MVPA were significant predictors of TEE among Daasanach participants (Figure 4; Table 4).

### 3.2. Water turnover

Across Daasanach communities, water turnover (litres/day) was positively correlated with TEE (Figure 5; Table 5) and more weakly correlated with FFM in multiple linear regression models of FFM, FM, sex and/or age (Figure 5; Table 5). As inferred from adjusted R2, Daasanach water turnover was better modelled as a function of TEE (R^2^ =0.7) and TEE + community (R^2^=0.28) than as a function of FFM, FM, and sex and/or age (R^2^ = 0.18–0.15). Models including MVPA as a predictor of water turnover were not significant (Table 5). When testing for differences in water turnover between Daasanach communities, community was not a significant predictor of water turnover in models of turnover as a function of community, TEE, and/or age (Table 5); there were no community level differences in FFM-adjusted or TEE-adjusted Daasanach water turnover (Figure 6).

When testing differences in water turnover between populations, both absolute Daasanach water turnover (mean: 7.54 litres/day men, 7.46 litres/day women) and TEE-adjusted water turnover rates were significantly higher (Tukey’s post-hoc *P* < 0.05) for Daasanach men and women than Hadza men and women and Shuar women (Figure 7; see figure legend for Hadza and Shuar mean water turnover values). For both Daasanach men and women, mean and TEE-adjusted water turnover were not significantly different from the mean and TEE-adjusted turnover of Shuar men (*p* > 0.30 for all comparisons, mean Shuar men turnover = 8.91 litres/day; Figure 7). Absolute mean Daasanach water turnover was also higher than mean values reported for a worldwide sample of adults (4.3 litres/day men, 3.4 litres/day women; Yamada et al. 2022). Relative to Daasanach, population (Shuar, Hadza) was a significant negative predictor of water turnover in multiple linear regression models of turnover as a function of both TEE and FFM + FM (Table 6, Figure 8). As inferred from adjusted R^2^, water turnover across small scale societies was slightly better modelled as a function of TEE, population, sex and/or age (R^2^ = 0.66) than as a function of FFM, FM, population, sex, and/or age (R^2^ = 0.65–0.64; Table 6).

## 4. Discussion

This study aimed to understand the energy and water needs of a semi-nomadic pastoralist population and test a) how these needs vary across communities with different levels of pastoralist activity and market integration and b) how they compare to other small-scale populations with high activity levels living in different climatic environments. We found that TEE and water turnover were not significantly different between Daasanach communities with different levels of market integration. Although Daasanach TEE was similar to that of other small-scale populations, water turnover was significantly higher in Daasanach communities than Shuar and Hadza. These data provide insights into the physiology of a population living a demanding lifestyle in an arid climate and highlight potential adaptations to extreme environments and water stress.

**Figure 2.**
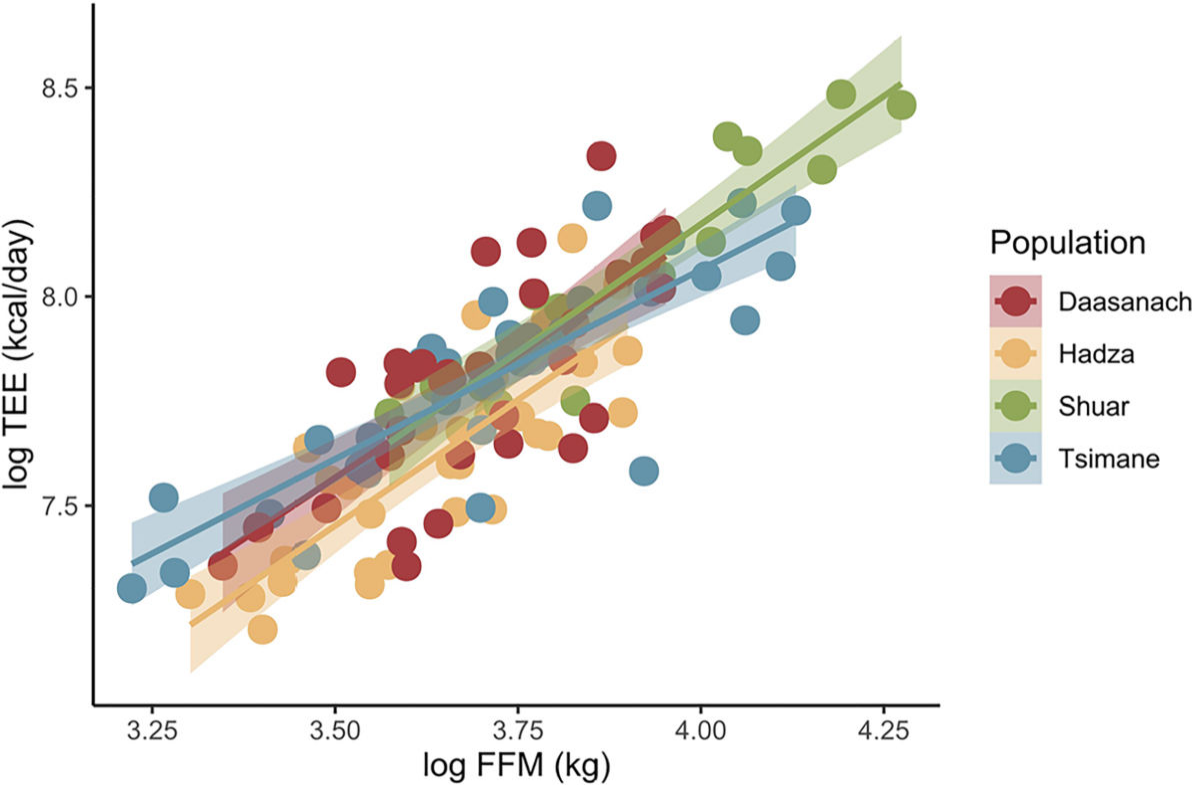
TEE as a function of FFM across populations. Daasanach TEE is no higher than that expected given lean mass compared to other small-scale societies.

**Figure 3.**
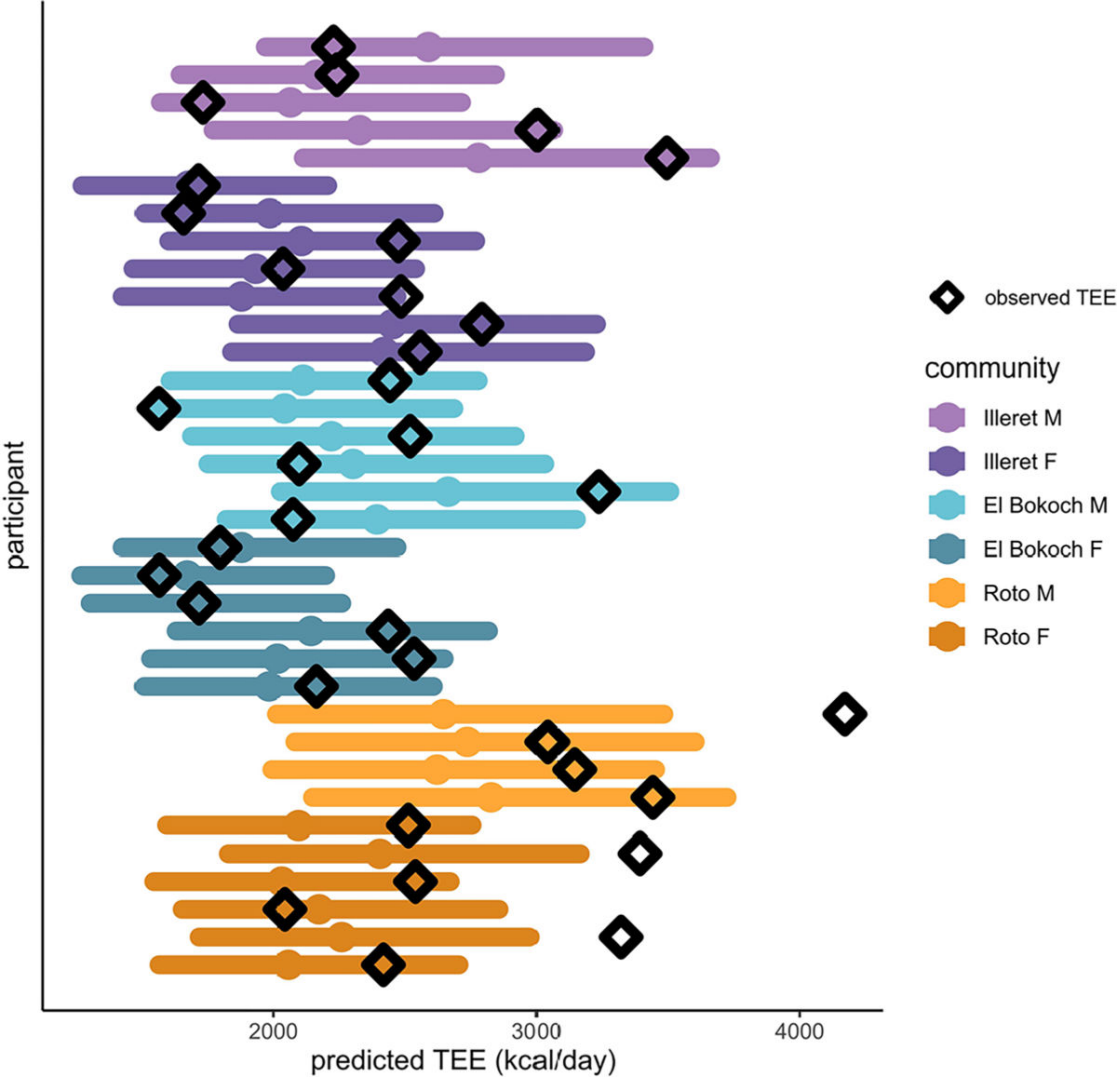
Predicted TEE (solid circle) for each Daasanach participant given FFM, FM, age, and sex based on linear models from Pontzer et al. 2021. All but three observed Daasanach TEEs (open diamonds) fall within the 95% prediction interval (coloured horizontal bars) for a worldwide sample of over 6000 adults.

The similar FFM-adjusted TEE of the Daasanach population and industrialised populations is compatible with the hypothesis that modern human TEE is constrained under an energetic ceiling (Pontzer 2018; Pontzer and McGrosky 2022). Although activity data was only collected from 10 Daasanach adults, 6 of the sampled Daasanach took more steps than the mean number of steps (5117) taken by American adults who participated in the America on the Move study (Bassett et al. 2010) and half of the sampled Daasanach took more steps than the 6886 steps averaged by Americans who participated in an 8-week physical activity challenge (Berko et al. 2016). Since many Daasanach also engage in physical activity that may not be captured by accelerometry (e.g. lifting heavy objects, digging wells), recorded steps per day and mean daily minutes of MVPA may underestimate physical activity level and/or be an imperfect proxy for activity energy expenditure. This suggests that despite an often physically active lifestyle, most Daasanach adults expend no more energy than adults in industrialised populations after adjusting for age, sex, fat mass, and fat free mass (Figure 2, Figure 3).

**Figure 4.**
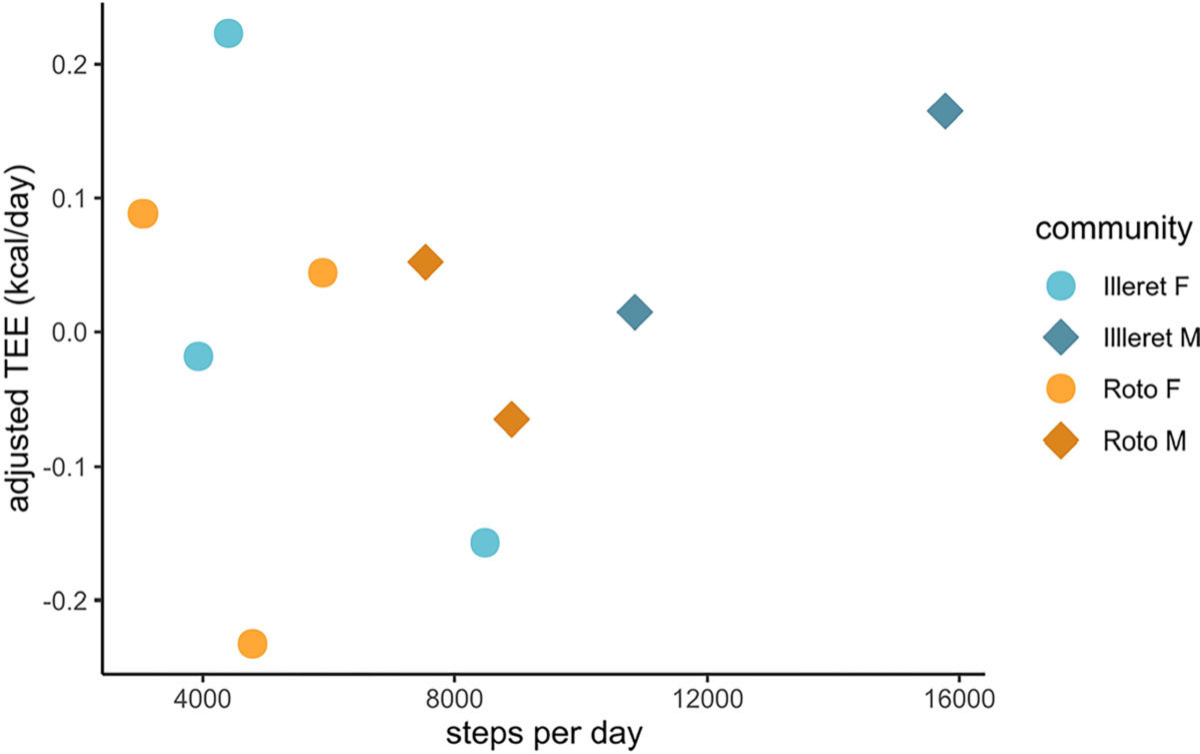
Daasanach TEE adjusted for FM and FFM as a function of mean steps per day. Adjusted TEE is not significantly correlated with mean steps per day.

**Figure 5.**
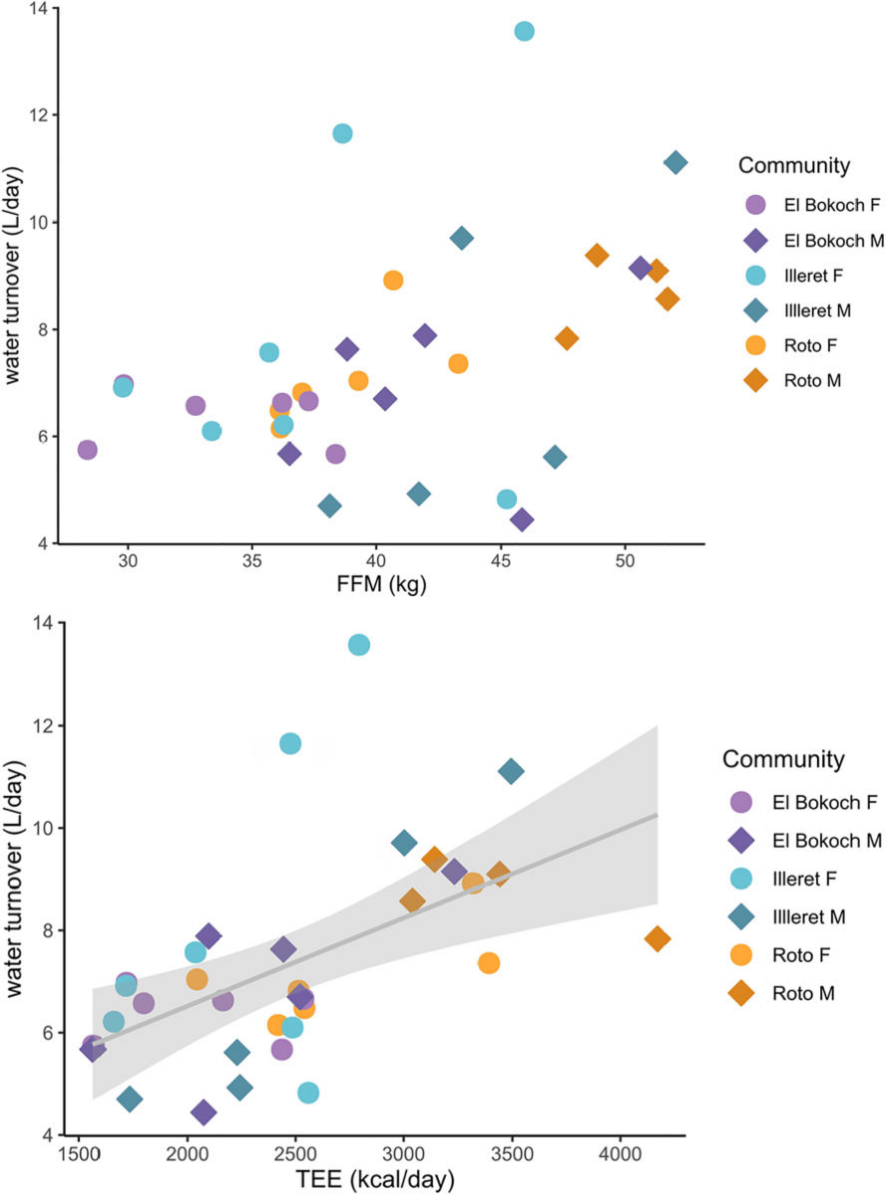
Daasanach water turnover. FFM(top panel) and TEE (bottom panel) are weakly positively correlated (adj. R^2^ < 0.30) with water turnover.

**Figure 6.**
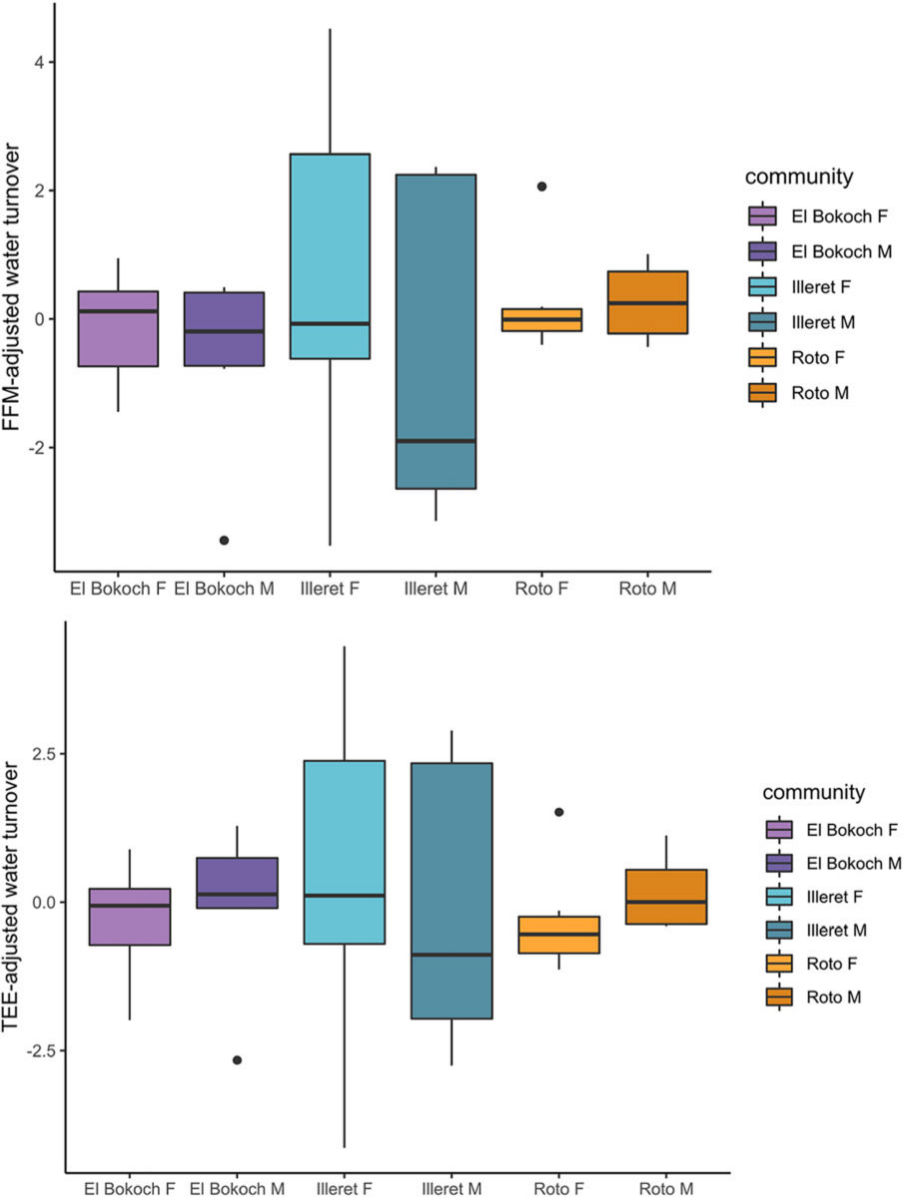
Daasanach water turnover. There are no differences in FFM-adjusted (top panel) and TEE-adjusted (bottom panel) turnover between Daasanach communities.

Although activity does not appear to be a predictor of total energy expenditure among Daasanach as a group (Figure 4), additional data are needed to explore potential short-term increases in energy expenditure as a result of lifestyle or physical activity demands. Three Daasanach individuals of the fora community of Roto exhibited TEEs higher than predicted from Pontzer et al. (2021)’s large dataset. Given the unique challenges of life in Roto–limited access to market resources, the potential for significant travel with livestock herds, and increased food and water stress relative to the more market-integrated Daasanach communities in Illeret and El Bokoch–it is possible that TEE in these individuals may be linked to the physical and physiological challenges of living in the remote environment of the fora. TEE increases substantially in response to physical activity levels and demands on short timescales (Thurber et al. 2019), so it is possible that the 3 high-TEE individuals were engaged in particularly demanding activities during the measurement week. Unfortunately, activity data were not collected from the high-TEE individuals. More work is also needed on movement patterns and activity levels of Daasanach individuals living in the fora, particularly as herding communities may seasonally adjust movement and activity as vegetation patterns shift. Observational data from Roto suggest that Daasanach in this community engage in high levels of physical activity that may not be captured by mean steps per day, which may necessitate trade-offs in energy allocation away from other demands such as immune responses and reproduction (in adults) or growth (in children) (Pontzer and McGrosky 2022). Additional data on physical activity and immune activity from Daasanach individuals across the life course are necessary to test these hypotheses.

Interestingly, even when accounting for age and fat mass, Daasanach women had higher fat free mass-adjusted TEE than Daasanach men (Table 2). Additional data on both physical activity, particularly demanding activities that may not be accurately captured by accelerometry (e.g. building houses, lifting 5- to 20-litre water jugs, collecting and carrying firewood) and immune activity are necessary to explore potential correlates of women’s elevated TEE.

FM is a predictor of TEE in larger samples of adults (Pontzer et al. 2021). The lack of significance of FM in many Daasanach models (Table 2) may be an artefact of the small Daasanach sample size and/or Daasanach body composition. All Daasanach participants had relatively low body fat (mean: 14 ± 6% men, 25 ± 8% women; population range: 4.5–37.6%), and it is likely that the larger Pontzer et al. (2021) sample included individuals spanning a wider range of body fat masses and percentages. Although age was not included as a co-variate in best-fitting models of Daasanach TEE (as inferred from adj. R2), age was a weak significant predictor of TEE in some models (Table 2), which could point to age-linked decreases in TEE. Almost all participants were between the ages of 20 and 40 (only 4 were over 40), so additional data are needed to establish whether age-linked decreases in adjusted TEE similar to those observed by Pontzer et al. (2021) occur among Daasanach.

**Figure 7.**
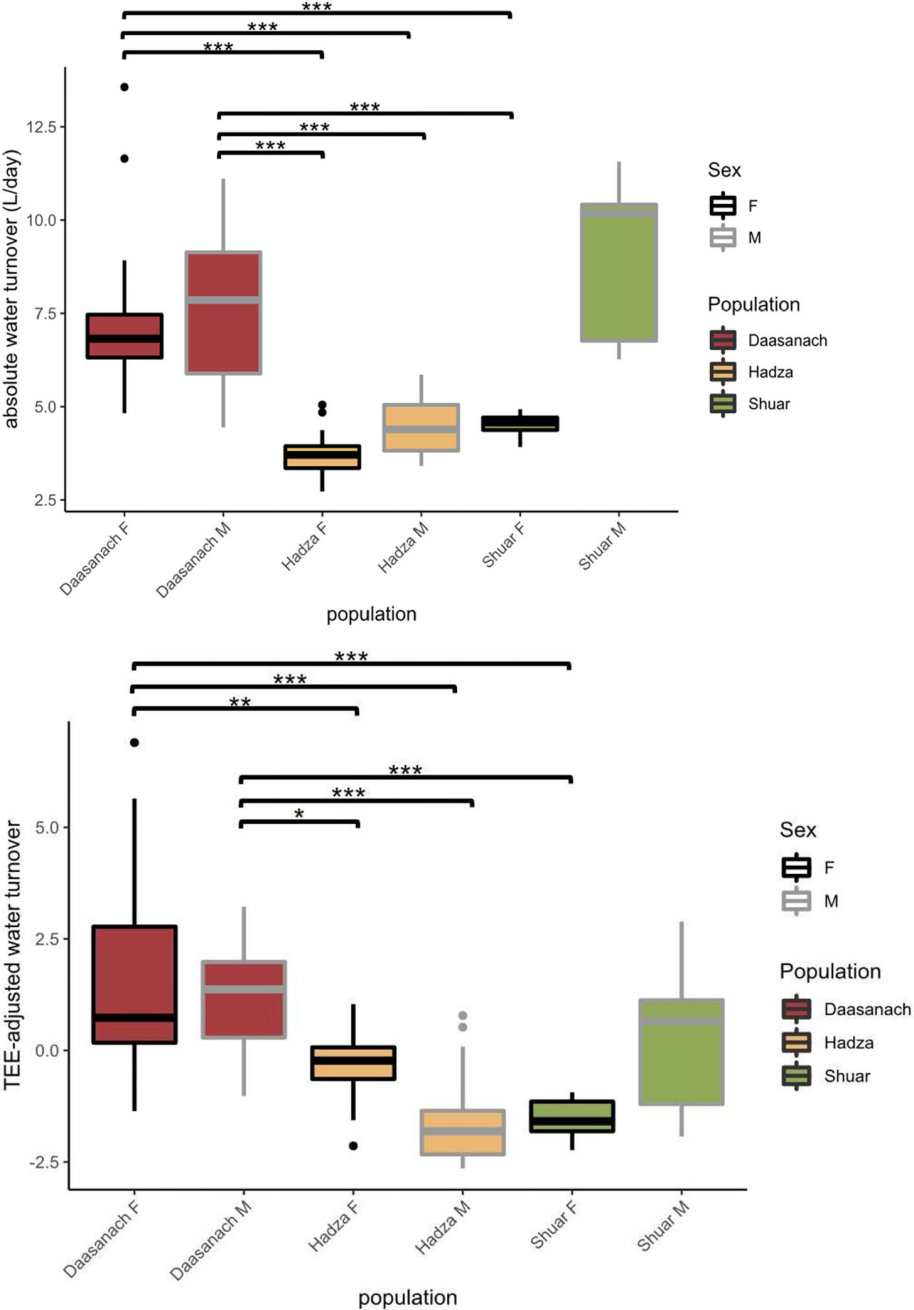
Absolute (top) and TEE-adjusted (bottom) water turnover across populations (***p < 0.001; **p < 0.01; *p < 0.05). Both Daasanach men (grey outline) and women (black outline) have higher water turnover rates than Hadza men, Hadza women and Shuar women (*p* < 0.05). Absolute and TEE-adjusted water turnover is not significantly different between Shuar men and both Daasanach men (absolute *p* = 0.49; TEE-adj. *p* = 0.79) and Daasanach women (absolute *p* = 0.36; TEE-adj. *p* = 0.30). Absolute and TEE-adjusted water turnover is not significantly different between Daasanach men and women (absolute *p* = 0.99; TEE-adj. *p* = 0.97). Although significance levels are not included in the plot, Shuar men’s TEE-adjusted water turnover is also significantly higher (*p* < 0.05; Tukey’s post-hoc test) than that of Shuar women and Hadza men. Hadza mean absolute water turnover: 3.74 litres/day women, 4.43 litres/day men; Shuar mean absolute water turnover: 8.91 litres/day men, 4.52 litres/day women.

**Figure 8.**
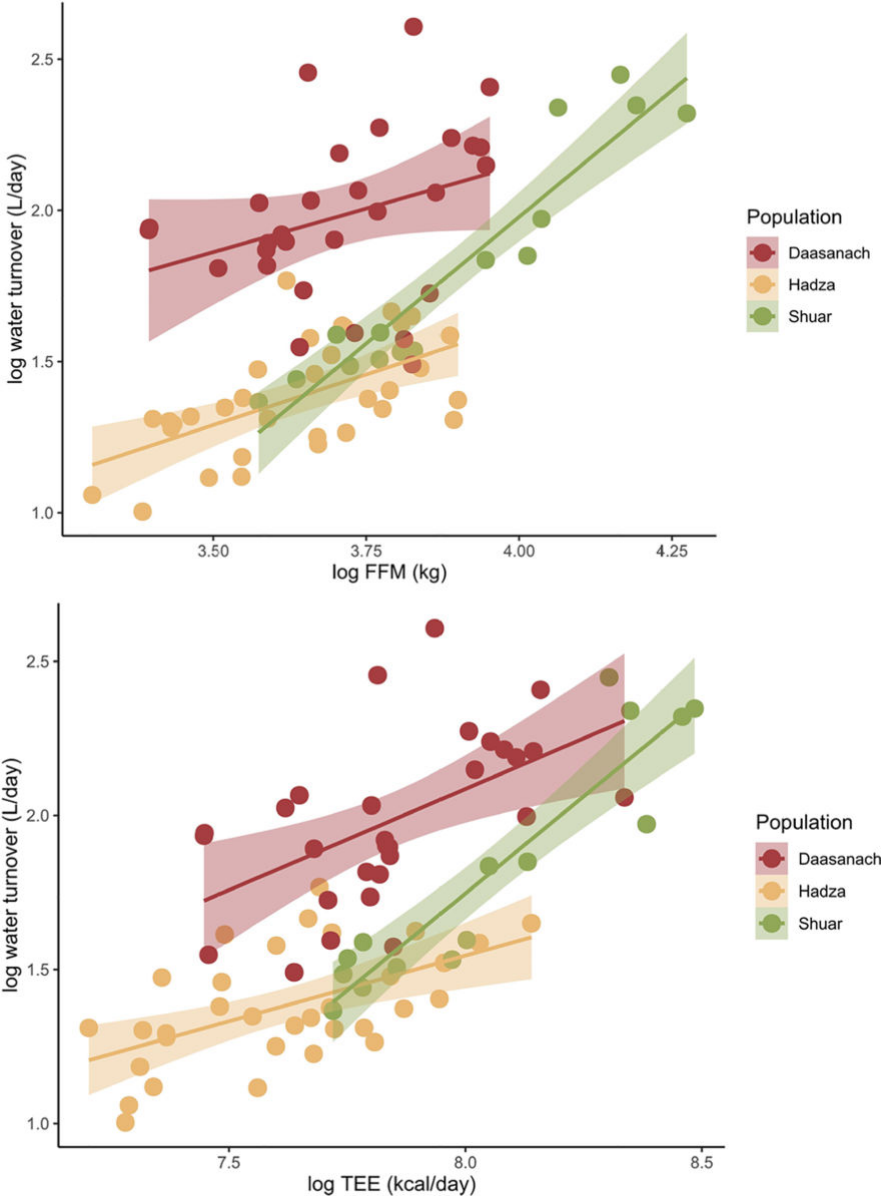
Daasanach water turnover at a given FFM (top panel) and TEE (bottom panel) is higher than that of other populations.

The positive correlation between Daasanach water turnover and TEE and FFM was expected due to the link between metabolic water production and metabolic rate; higher metabolic rates produce more metabolic water (Shimamoto and Komiya 2000). The significantly higher water turnover among Daasanach than among most other documented contemporary populations (Figure 7, Figure 8) and approximately twice that of a worldwide sample of adults (Yamada et al. 2022) may be a product of their lifestyle, environment, and accompanying water demands; comparably high water turnover among Shuar men is likely due to their cultural hydration strategy of consuming a home-made traditional fermented beverage (chicha) (Christopher et al. 2019).

Daasanach lifestyles are physically demanding and high levels of physical activity lead to increased sweating and respiration, which can contribute to body water loss. Human water needs can easily top 6L/day with physical activity in hot, dry environments as moisture is lost to sweat (Sawka et al. 2005). Deuterium-based methods do not directly measure water intake, which represents about 80–85% of water turnover volume (Raman et al. 2004); reducing mean Daasanach water turnover (7.5 L/day) by 20% yields a daily water intake from fluid and food sources of ~6 litres.

While a “hyper-hydrated” lifestyle may escm difficult to support in a water-limited environment, low urinary specific gravity values among adults in this population suggest that many Daasanach individuals could be well-hydrated (Bethancourt et al. 2021). Maintaining adequate hydration may be an economic and cultural priority; Daasanach have a daily habit of drinking tea, and water sharing is an integral component of Daasanach culture and a critical hydration strategy (Bethancourt et al. 2021; Ford et al. 2023). Forthcoming work that incorporates additional water turnover data among Daasanach adults will further explore the relationship between water turnover, lifestyle, and climate.

Alternatively, high water turnover may be related to kidney function. Acute kidney injury, such as that caused by heat stress, hyperthermia, dehydration, and malaria, can increase the risk of chronic kidney disease (Koopmans et al. 2015; Conroy et al. 2019; Batte et al. 2021; Namazzi et al. 2022). Chronic kidney disease is characterised by the destruction of nephrons; remaining nephrons hypertrophy and engage in hyperfiltration to compensate for damaged nephrons and maintain sufficient total kidney filtration rates, but failing kidneys eventually lose the ability to maximally concentrate urine (Agaba et al. 2012; Sands and Layton 2014). Thus, the kidneys must excrete more water to eliminate waste solutes, which increases daily water needs. Individuals with chronic kidney disease may therefore present with both high daily water needs and dilute urine. Prior work has demonstrated that 30% of Daasanach adults had hyper-dilute urine, an indicator of kidney dysfunction, which was associated with the salinity of drinking water (Rosinger et al. 2021). The high level of salt consumed in water may have important implications for body water homeostasis in this population, particularly as many members of the community consume over 6 litres per day. This study’s findings of high water turnover among Daasanach, coupled with a high prevalence of hyper-dilute urine (Bethancourt et al. 2021), suggests that kidney issues may underpin aspects of water balance and daily water requirements in this population (Rosinger et al. 2021), but more work is needed to untangle these relationships.

## 5. Limitations and conclusions

The moderate sample size, limited number of participants with empirically measured physical activity, and limited scope of our data collection constrain the conclusions we can draw from this analysis. Given the small sample size, increasing the number of covariates in our linear regression models reduces the power of the models. We also note that we assumed a food quotient of 0.86 in our energy expenditure calculations, which is common practice for populations who lack detailed dietary assessments and assumes that fats account for ~30% of the daily energy intake (IAEA 2009). If the fat content of the Daasanach diet is lower, as seen in some other subsistence communities (Pontzer et al. 2018), then their calculated TEEs will be in the order of 3% to 5% lower than we report here. Further work is needed to accurately assess Daasanach diet composition.

With these caveats in mind, TEE does not appear to correlate with daily activity among Daasanach participants in this sample. Consistent with constrained energy expenditure models (e.g. Pontzer 2018), the Daasanach sample as a whole does not show elevated TEE compared to other populations. Additional measures, however, are needed to confirm these findings. The data in this analysis were collected during one part of the year (late June through early July in both years), and thus we are not able to assess possible seasonal fluctuations in water turnover or TEE. Future work should explore both TEE and water turnover during different times of the year, particularly as climate change continues to affect this population living in one of the hottest land areas on Earth.

Additional physical activity data are required to more robustly test the hypothesis that Daasanach TEEs are elevated during periods of high physical activity, as well as to explore potential trade-offs between physical activity and growth (in children) or immune activity (in both children and adults) that would be predicted under constrained energy expenditure models (Pontzer 2018; Pontzer and McGrosky 2022). Observational data suggest that accelerometry-derived step counts and activity minutes (which are determined by displacement) may be insufficient to fully capture the range of physical activities performed by Daasanach participants (e.g. lifting objects, well-digging, climbing steep terrain). This may limit the usefulness of accelerometry as a proxy for activity levels and thus, activity energy expenditure in this, and other, populations. Other proxies for activity level, such as heart rate, may be more useful tools for estimating physical activity level or activity energy expenditure. Finally, this analysis focused on adults between the ages of 18 and 60 years, a period when FFM-adjusted TEE is stable (Pontzer et al. 2021) and repeatable (Rimbach et al. 2022). A wider age range is needed to assess potential age effects on Daasanach metabolic physiology and to identify which effects, if any, may be linked to the community’s transition away from pastoralism towards a more market-integrated lifestyle.

Nonetheless, our findings that habitual, population-level total energy expenditure does not appear to be linked to the degree of pastoral activity, physical activity, and lifestyle either between or within populations, reinforces the role pastoralism plays as a successful adaptive strategy to meet animal pasture and water needs in marginal environments. Despite their typical energy requirements, the high daily water requirements of Daasanach living in the hot, dry Turkana Basin has further implications for worldwide water needs and kidney health as climate change and drought expand the global extent of hot, dry environments.

## Supplementary Material

supplemental information

## Figures and Tables

**Table 1.** Anthropometrics, TEE and water turnover of Daasanach participants from the El Bokoch, Illeret, and Roto communities.

	Age	Sex	BM (kg)	TEE (kcal/day)	FFM (kg)	Fat %	water turnover (L/day)	mean daily steps	mean daily mins MVPA	ND/NO	kD	kO	ND	NO

El Bokoch (*n* = 12)	38	F	48	2164	36.2	24.7	6.63			1.043	0.2269	0.2604	1539.2	1476.1
	38	F	55	2536	37.3	32.7	6.66			1.033	0.2225	0.2593	1577.7	1526.7
	23	F	50	2437	38.4	22.8	5.67			1.036	0.1839	0.2174	1625.4	1569.6
	25	F	45	1717	29.8	33.7	6.97			1.046	0.2893	0.3240	1270.0	1214.1
	22	F	39	1566	28.4	26.6	5.75			1.036	0.2515	0.2839	1203.6	1161.4
	22	F	44	1797	32.7	26.0	6.57			1.033	0.2501	0.2823	1384.8	1340.3
	54	M	52	2075	45.8	11.9	4.44			1.048	0.1197	0.1431	1953.6	1863.8
	37	M	59	3235	50.6	14.8	9.15			1.028	0.2257	0.2609	2136.4	2079.0
	32	M	50	2098	42.0	16.1	7.89			1.029	0.2344	0.2640	1772.2	1722.2
	32	M	50	2521	40.4	19.3	6.70			1.018	0.2083	0.2422	1695.0	1665.3
	34	M	44	1564	36.5	17.6	5.67			1.038	0.1930	0.2181	1548.8	1491.5
	40	M	50	2444	38.8	21.7	7.63			1.037	0.2441	0.2795	1645.6	1587.6

llleret (*n* = 12)	31	F	57	2559	45.2	21.1	4.82	8483	61	1.028	0.1331	0.1619	1908.8	1856.9
	26	F	65	2794	45.9	29.0	13.56	3931	18	1.032	0.3678	0.4067	1942.4	1882.9
	30	F	46	2486	33.4	27.3	6.10	4409	17	1.041	0.2267	0.2663	1418.0	1361.8
	39	F	57	2037	35.7	37.6	7.57			1.040	0.2633	0.2969	1514.9	1456.8
	36	F	50	2476	38.7	22.2	11.65			1.035	0.3750	0.4155	1636.9	1582.0
	35	F	52	1658	36.3	30.1	6.21			1.032	0.2134	0.2404	1534.1	1486.0
	36	F	47	1715	29.8	36.6	6.91			1.050	0.2865	0.3212	1271.2	1210.1
	35	M	56	3494	52.0	7.3	11.11	10845	56	1.036	0.2654	0.3034	2204.5	2128.3
	45	M	49	3003	43.5	12.2	9.71	15776	33	1.035	0.2779	0.3172	1840.5	1777.6
	50	M	45	1732	38.1	14.5	4.70			1.037	0.1532	0.1779	1616.9	1558.6
	56	M	57	2242	41.7	26.8	4.92			1.027	0.1474	0.1755	1759.8	1714.1
	22	M	55	2229	47.2	13.9	5.61			1.042	0.1475	0.1728	2004.8	1923.6

Roto (*n* = 10)	20	F	46	2419	36.2	20.7	6.15	5905	32	1.028	0.2122	0.2481	1525.7	1484.8
	31	F	45	3320	40.7	10.2	8.92	3059	13	1.044	0.2715	0.3158	1730.5	1658.3
	28	F	49	2044	39.3	19.6	7.04	4790	13	1.054	0.2207	0.2507	1679.0	1593.6
	30	F	43	2541	36.1	15.8	6.48			1.063	0.2201	0.2579	1549.6	1458.0
	20	F	51	3392	43.3	14.9	7.36			1.060	0.2090	0.2497	1856.0	1751.3
	20	F	47	2514	37.0	21.7	6.82			1.041	0.2285	0.2655	1572.4	1510.2
	25	M	54	3441	51.3	4.5	9.10	8907	66	1.041	0.2201	0.2564	2177.1	2092.3
	34	M	55	3143	48.9	10.8	9.39	7538	21	1.041	0.2387	0.2741	2040.1	1987.9
	34	M	59	3041	51.7	12.5	8.57			1.040	0.2057	0.2380	2195.3	2111.2
	22	M	52	4172	47.7	7.8	7.83			1.041	0.2038	0.2482	2024.2	1944.0

ND: deuterium dilution space; NO: oxygen-18 dilution space; kD: deuterium elimination rate; kO:oxygen-18 elimination rate.

**Table 2.** Daasanach TEE (kcal/day) model coefficients and intercepts. Reference community is Illeret. Bolded cells are significant.

				InTEE ~					
	
	InFFM + lnFM + sex	InFFM + InFM + age + sex	lnFFM + lnFM + sex + community	lnFFM + lnFM + age + sex + community	InFFM + sex	InFFM + age	InFFM + lnFM + age	InFFM + lnFM	InFFM
									
	est. (se)	est. (se)	est. (se)	est. (se)	est. (se)	est. (se)	est. (se)	est. (se)	est. (se)

intercept	3.35*** **(0.88)**	3.40*** **(0.89)**	3.37** **(0.95)**	3.42** **(0.97)**	2.30** **(0.81)**	3.49*** **(0.64)**	4.03*** **(0.90)**	4.28*** **(0.94)**	3.46*** **(0.69)**
InFFM (kg)	1.33*** **(0.22)**	1.32*** **(0.23)**	1.26*** **(0.25)**	1.26*** **(0.25)**	1.51*** **(0.22)**	1.23*** **(0.18)**	1.12*** **(0.22)**	1.01*** **(0.23)**	1.17*** **(0.19)**
InFM (kg)	−0.16* **(0.07)**	−0.14 (0.08)	−0.09 (0.09)	−0.09 (0.09)	-	-	−0.06 (0.07)	−0.09 (0.08)	
age (yrs)	-	−0.002 (0.003)	-	−0.001 (0.004)	-	−0.007* **(0.003)**	−0.007* **(0.003)**	-	-
sex-M	−0.22** **(0.07)**	−0.19* **(0.08)**	−0.17 (0.09)	−0.15 (0.10)	−0.17* **(0.07)**	-	-	-	-
community-EI Bokoch	-	-	0.01 (0.07)	0.003 (0.07)	-	-	-	-	-
community-Roto	-	-	0.11 (0.08)	0.10 (0.09)	-	-	-	-	-
adj. R^2^	0.65	0.64	0.64	0.63	0.60	0.60	0.59	0.55	0.54

*** *p* < 0.001

** *p* < 0.01

* *p* < 0.05

Se: standard error in parentheses

**Table 3.** Coefficients and intercepts for multiple regression model of TEE across small-scale societies (Daasanach, Hadza, Shuar, Tsimane’), compared to Pontzer et al. (2021). Reference population is Daasanach. Bolded cells are significant.

	InTEE ~	
	
	InFFM + InFM + age + sex + popuIation	InFFM + InFM + age + sex
		
		Pontzer et aI. (2021)
		
		*Adults 20–60 years*

	est. (se)	est. (se)
intercept	4.03*** **(0.39)**	4.70*** **(0.06)**
InFFM (kg)	1.04*** **(0.11)**	0.84*** **(0.02)**
InFM (kg)	−0.014 (0.026)	−0.02*** **(0.01)**
age (yrs)	−0.001 (0.001)	−0.002*** **(<0.001)**
sex-M	0.0001 (0.04)	0.01 (0.01)
population-Hadza	−0.11** **(0.04)**	
population-Shuar	0.05 (0.05)	
population-Tsimane’	0.02 (0.04)	
adj. R^2^	0.73	0.65

*** *p* < 0.001

** *p* < 0.01

* *p* < 0.05

Se: standard error

**Table 4.** Daasanach TEE as a function of activity model coefficients. Either steps per day or minutes of moderate and vigorous physical activity (MVPA) per day are used to infer activity level. Bolded cells are significant.

InTEE ~							
	
	InFFM + InFM + MVPA	InFFM + lnFM + steps	InFFM + InFM + age + steps	InFFM + InFM + age + MVPA	InFFM + InFM + sex + MVPA	lnFFM + lnFM + sex + steps	InFFM + InFM + age + sex + steps
							
		est. (se)	est. (se)	est. (se)	est. (se)	est. (se)	est. (se)

intercept	5.98** **(1.42)**	6.19** **(1.37)**	5.97** **(1.41)**	6.05* **(1.53)**	6.10* **(1.73)**	6.49* **(1.64)**	6.17* **(1.76)**
InFFM (kg)	0.61 (0.37)	0.55 (0.35)	0.56 (0.35)	0.56 (0.41)	0.57 (0.48)	0.45 (0.44)	0.50 (0.46)
InFM (kg)	−0.15 (0.08)	−0.14 (0.01)	−0.15 (0.08)	−0.14 (0.09)	−0.14 (0.11)	−0.12 (0.11)	−0.13 (0.12)
age (yrs)	-	-	0.01 (0.001)	0.003 (0.007)	-	-	0.008 (0.01)
sex-M	-	-	-	-	0.02 (0.15)	0.09 (0.22)	0.06 (0.02)
mean steps/day	-	<0.001 (<0.001)	<0.001 (<0.001)	-	-	<0.001 (<0.001)	−0.16 (<0.001)
mean daily mins MVPA	−0.001 (0.003)	-	-	−0.001 (0.002)	−0.001 (0.003)	-	-
adj. R^2^	0.51	0.49	0.48	0.44	0.42	0.41	0.36

*** *p* < 0.001

** *p* < 0.01

* *p* < 0.05

Se: standard error

**Table 5.** Daasanach water turnover (litres/day) model coefficients and intercepts. Reference community Illeret. Bolded cells significant.

					water turnover ~				
	
	TEE + community	TEE	FFM + FM + sex	FFM + FM + age	FFM + FM + sex + age	FFM + FM	FFM + FM + age + community	FFM + FM + sex + MVPA	TEE + MVPA
									
	est. (se)	est. (se)	est. (se)	est. (se)	est. (se)	est. (se)	est. (se)	est. (se)	est. (se)

intercept	**−3.01** * **(1.42)**	**3.07** * **(1.22)**	−1.47 (2.98)	0.14 (2.96)	−0.81 (3.19)	−0.53 (2.96)	1.46 (3.54)	−6.95 (6.42)	−0.88 (4.44)
TEE (kcal/day)	0.002** **(0.0006)**	0.002** **(0.0005)**	-	-	-	-	-	-	0.004 (0.002)
FFM (kg)	-	-	0.22** **(0.07)**	0.19** **(0.06)**	0.21** **(0.07)**	0.17** **(0.06)**	0.17* **(0.06)**	0.37 (0.18)	-
FM (kg)	-	-	0.05 (0.09)	0.12 (0.17)	0.08 (0.10)	0.10 (0.08)	0.10 (0.10)	0.18 (0.19)	-
age (yrs)	-	-	-	−0.05 (0.04)	−0.03 (0.04)	-	−0.05 (0.04)	-	-
sex-M	-	-	−1.28 (0.88)	-	−0.90 (1.07)	-	-	1.49 (2.36)	-
community-EI Bokoch	−0.72 (0.72)	-	-	-	-	-	−0.70 (0.83)	-	-
community-Roto	−1.24 (0.83)	-	-	-	-	-	−0.43 (1.06)	-	-
MVPA	-	-	-	-	-	-	-	−0.08 (0.04)	−0.05 (0.04)
adj. R^2^	0.28	0.27	0.19	0.18	0.18	0.16	0.15	0.45	0.28

*** *p* < 0.001

** *p* < 0.01

* *p* < 0.05

Se: standard error

**Table 6.** Water turnover as a function of TEE and FFM across populations. Bolded cells significant.

					water turnover~			
	
	TEE + population	TEE + sex + population	TEE + age + population	TEE + age + sex + population	FFM + FM + sex + population	FFM + FM + sex + age + population	FFM + FM + age + population	FFM + FM + population
								
	est. (se)	est. (se)	est. (se)	est. (se)	est. (se)	est. (se)	est. (se)	est. (se)

intercept	2.87*** **(0.63)**	2.74*** **(0.67)**	2.95** **(0.78)**	2.79** **(0.84)**	−0.61 (1.20)	−0.29 (1.30)	0.51 (1.13)	0.19 (1.08)
TEE (kcal/day)	0.002*** **(0.0002)**	0.002*** **(0.0003)**	0.002*** **(0.0002)**	0.002*** **(0.0003)**	-	-	-	-
FFM (kg)	-	-	-	-	0.19*** **(0.31)**	0.19*** **(0.03)**	0.16*** **(0.02)**	0.16*** **(0.02)**
FM (kg)	-	-	-	-	0.05 (0.04)	0.05 (0.04)	0.07 (0.04)	0.07 (0.04)
age (yrs)	-	-	−0.002 (0.01)	−0.001 (0.01)	-	−0.008 (0.01)	−0.01 (0.01)	-
sex-M	-	−0.20 (0.37)	-	−0.20 (0.38)	−0.70 (0.48)	−0.63 (0.50)	-	-
population-Hadza	−2.60*** **(0.35)**	−2 59*** **(0.36)**	−2 59*** **(0.35)**	−2.55*** **(0.36)**	−2.81*** **(0.36)**	−2.76*** **(0.37)**	−2.76*** **(0.37)**	−2.83*** **(0.36)**
population-Shuar	−2.18*** **(0.46)**	—2.24*** **(0.48)**	−2.17*** **(0.47)**	−2.23*** **(0.49)**	−3.01*** **(0.58)**	−3.00*** **(0.58)**	−2.85*** **(0.57)**	−2.84*** **(0.57)**
adj. R^2^	0.66	0.66	0.66	0.65	0.65	0.64	0.64	0.64

*** *p* < 0.001

** *p* < 0.01

* *p* < 0.05

Se: standard error

## References

[R1] El Agaba, Rohrscheib M, Tzamaloukas AH. 2012. The renal concentrating mechanism and the clinical consequences of its loss. Niger Med J. 53(3):1–15. doi: 10.4103/0300-1652.104376. 23293407 PMC3531026

[R2] Bassett DRJ, Wyatt HR, Thompson H, Peters JC, Hill JO. 2010. Pedometer-measured physical activity and health behaviors in U.S. Adults. Med Sci Sports Exerc 42(10):1819–1825. doi: 10.1249/MSS.0b013e3181dc2e54. 20305579 PMC2927728

[R3] Batte A, Berrens Z, Murphy K, Mufumba I, Sarangam ML, Hawkes MT, Conroy AL. 2021. Malaria-associated acute kidney injury in African Children: prevalence, pathophysiology, impact, and management challenges. int J Nephrol Renovasc Dis. 14:235–253. doi: 10.2147/IJNRD.S239157. 34267538 PMC8276826

[R4] Berko J, Goetzel RZ, Roemer EC, Kent K, Marchibroda J. 2016. Results from the bipartisan policy center’s CEO council physical activity challenge to American Business. J Occup Environ Med. 58(12):1239–1244. doi: 10.1097/JOM.0000000000000897. 27930485 PMC5181119

[R5] Bethancourt HJ, Swanson ZS, Nzunza R, Huanca T, Conde E, Kenney WL, Young SL, Ndiema E, Braun D, Pontzer H, 2021. Hydration in relation to water insecurity, heat index, and lactation status in two small-scale populations in hot-humid and hot-arid environments. Am J Hum Biol. 33(1):e23447. doi: 10.1002/ajhb.23447. PMC8829588 32583580

[R6] Bethancourt HJ, Swanson ZS, Nzunza R, Young SL, Lomeiku L, Douglass MJ, Braun DR, Ndiema EK, Pontzer H, Rosinger AY. 2022. The co-occurrence of water insecurity and food insecurity among Daasanach pastoralists in northern Kenya. Public Health Nutr. 26(3):1–11. doi: 10.1017/S1368980022001689. PMC9989708 35941080

[R7] Choi L, Beck C, Liu Z, Moore R, Matthews C, Buchowski M. 2021. PhysicalActivity: Process accelerometer data for physical activity measurement. R package version 0.2–4. https://CRAN.R-project.org/package=PhysicalActivity

[R8] Christopher L, Madimenos FC, Bribiescas RG, Urlacher SS, Snodgrass JJ, Sugiyama LS, Pontzer H. 2019. High energy requirements and water throughput of adult Shuar forager-horticulturalists of Amazonian Ecuador. Am J Hum Biol. 31(2):e23223. doi: 10.1002/ajhb.23223. 30801886

[R9] Conroy AL, Opoka RO, Bangirana P, Idro R, Ssenkusu JM, Datta D, Hodges JS, Morgan C, John CC. 2019. Acute kidney injury is associated with impaired cognition and chronic kidney disease in a prospective cohort of children with severe malaria. BMC Med. 17(1):98. doi: 10.1186/s12916-019-1332-7. 31109328 PMC6528242

[R10] Dugas LR, Harders R, Merrill S, Ebersole K, Shoham DA, Rush EC, Assah FK, Forrester T, Durazo-Arvizu RA, Luke A. 2011. Energy expenditure in adults living in developing compared with industrialized countries: a meta-analysis of doubly labeled water studies. Am J Clin Nutr. 93(2):427–441. doi: 10.3945/ajcn.110.007278. 21159791 PMC3021434

[R11] Ford LB, Bethancourt HJ, Swanson ZS, Nzunza R, Wutich A, Brewis A, Young S, Almeida DM, Douglass M, Ndiema EK, 2023. Water insecurity, water borrowing and psychosocial stress among Daasanach pastoralists in northern Kenya. Water Int. 48(1):63–86. doi: 10.1080/02508060.2022.2138050. 38800511 PMC11126231

[R12] Gurven MD, Trumble BC, Stieglitz J, Yetish G, Cummings D, Blackwell AD, Beheim B, Kaplan HS, Pontzer H. 2016. High resting metabolic rate among Amazonian forager-horticulturalists experiencing high pathogen burden. Am J Phys Anthropol. 161(3):414–425. doi: 10.1002/ajpa.23040. 27375044 PMC5075257

[R13] IAEA. 2009. Assessment of Body Composition and Total Energy Expenditure in Humans Using Stable Isotope Techniques. Vienna: I.A.E.A.

[R14] Keadle SK, Shiroma EJ, Freedson PS, Lee I-M. 2014. Impact of accelerometer data processing decisions on the sample size, wear time and physical activity level of a large cohort study. BMC Public Health. 14(1):1210. doi: 10.1186/1471-2458-14-1210. 25421941 PMC4247661

[R15] Kleiner SM. 1999. Water: an essential but overlooked nutrient. J Am Diet Assoc. 99(2):200–206. doi: 10.1016/S0002-8223(99)00048-6. 9972188

[R16] Koopmans LC, van Wolfswinkel ME, Hesselink DA, Hoorn EJ, Koelewijn R, van Hellemond JJ, van Genderen PJJ. 2015. Acute kidney injury in imported Plasmodium falciparum malaria. Malar J. 14(1):523. doi: 10.1186/512936-015-1057-9. 26702815 PMC4690233

[R17] Luke A, Dugas LR, Ebersole K, Durazo-Arvizu RA, Cao G, Schoeller DA, Adeyemo A, Brieger WR, Cooper RS. 2009. Energy expenditure does not predict weight change in either Nigerian or African American women. Am J Clin Nutr. 89(1):169–176. doi: 10.3945/ajcn.2008.26630. 19056567 PMC2647711

[R18] Michaud DS, Spiegelman D, Clinton SK, Rimm EB, Curhan GC, Willett WC, Giovannucci EL. 1999. Fluid Intake and the Risk of Bladder Cancer in Men. N Engl J Med. 340(18):1390–1397. doi: 10.1056/NEJM199905063401803. 10228189

[R19] Mwamidi D, Renom JG, Fernandez-Llamazares Onrubia A, Burgas Riera D, Domínguez P, Cabeza-Jaimejuan MDM. 2018. Contemporary pastoral commons in East Africa as OECMs: a case study from the Daasanach community. Parks Int J Prot Areas Conserv. 24:79–88. doi: 10.2305/IUCN.CH.2018.PARKS-24-SIDMM.en.

[R20] Namazzi R, Batte A, Opoka RO, Bangirana P, Schwaderer AL, Berrens Z, Datta D, Goings M, Ssenkusu JM, Goldstein SL, 2022. Acute kidney injury, persistent kidney disease, and post-discharge morbidity and mortality in severe malaria in children: a prospective cohort study. eClinicalMedicine. 44:101292. doi: 10.1016/j.eclinm.2022.101292. PMC8850340 35198918

[R21] Noakes TD. 1998. Fluid and electrolyte disturbances in heat illness. Int J Sports Med. 19(S 2):S146–S149. doi: 10.1055/s-2007-971982. 9694423

[R22] Opiyo F 2013. Trend analysis of rainfall and temperature variability in arid environment of Turkana, Kenya. Environ Res J. 8:30–43.

[R23] Passey BH, Levin NE, Cerling TE, Brown FH, Eiler JM. 2010. High-temperature environments of human evolution in East Africa based on bond ordering in paleosol carbonates. Proc Natl Acad Sci U S A. 107(25):11245–11249. doi: 10.1073/pnas.1001824107. 20534500 PMC2895143

[R24] Pearle MS. 2001. Prevention of nephrolithiasis. Curr Opin Nephrol Hypertens. 10(2):203–209. doi: 10.1097/00041552-200103000-00008. 11224695

[R25] Pontzer H 2018. Energy constraint as a novel mechanism linking exercise and health. Physiology. 33(6):384–393. doi: 10.1152/physiol.00027.2018. 30303776

[R26] Pontzer H, Durazo-Arvizu R, Dugas LR, Plange-Rhule J, Bovet P, Forrester TE, Lambert EV, Cooper RS, Schoeller DA, Luke A. 2016. Constrained total energy expenditure and metabolic adaptation to physical activity in adult humans. Curr Biol. 26(3):410–417. doi: 10.1016/j.cub.2015.12.046. 26832439 PMC4803033

[R27] Pontzer H, McGrosky A. 2022. Balancing growth, reproduction, maintenance, and activity in evolved energy economies. Curr Biol. 32(12):R709–R719. doi: 10.1016/j.cub.2022.05.018. 35728556

[R28] Pontzer H, Raichlen DA, Wood BM, Emery Thompson M, Racette SB, Mabulla AZP, Marlowe FW. 2015. Energy expenditure and activity among Hadza hunter-gatherers. Am J Hum Biol. 27(5):628–637. doi: 10.1002/ajhb.22711. 25824106

[R29] Pontzer H, Raichlen DA, Wood BM, Mabulla AZP, Racette SB, Marlowe FW. 2012. Hunter-Gatherer energetics and human obesity. PLOS One. 7(7):e40503. doi: 10.1371/journal.pone.0040503. PMC3405064 22848382

[R30] Pontzer H, Wood BM, Raichlen DA. 2018. Hunter-gatherers as models in public health. Obes Rev. 19 Suppl 1(S1):24–35. doi: 10.1111/obr.12785. 30511505

[R31] Pontzer H, Yamada Y, Sagayama H, Ainslie PN, Andersen LF, Anderson LJ, Arab L, Baddou I, Bedu-Addo K, Blaak EE, 2021. Daily energy expenditure through the human life course. Science. 373(6556):808–812. doi: 10.1126/science.abe5017. 34385400 PMC8370708

[R32] Raman A, Schoeller DA, Subar AF, Troiano RP, Schatzkin A, Harris T, Bauer D, Bingham SA, Everhart JE, Newman AB, 2004. Water turnover in 458 American adults 40–79yr of age. Am J Physiol Renal Physiol. 286(2):F394–F401. doi: 10.1152/ajprenal.00295.2003. 14600032

[R33] Rimbach R, Yamada Y, Sagayama H, Ainslie PN, Anderson LF, Anderson LJ, Arab L, Baddou I, Bedu-Addo K, Blaak EE, 2022. Total energy expenditure is repeatable in adults but not associated with short-term changes in body composition. Nat Commun. 13(1):99. doi: 10.1038/s41467-021-27246-z. 35013190 PMC8748652

[R34] Roncal-Jimenez C, Lanaspa MA, Jensen T, Sanchez-Lozada LG, Johnson RJ. 2015. Mechanisms by which dehydration may lead to chronic kidney disease. Ann Nutr Metab. 66 Suppl 3(Suppl. 3):10–13. doi: 10.1159/000381239. 26088040

[R35] Rosinger AY. 2020. Biobehavioral variation in human water needs: how adaptations, early life environments, and the life course affect body water homeostasis. Am J Hum Biol. 32(1):e23338. doi: 10.1002/ajhb.23338. 31631450

[R36] Rosinger AY. 2023. Water needs, water insecurity, and human biology. Annu Rev Anthropol. 52(1):93–113. doi: 10.1146/annurev-anthro-052721-090331.

[R37] Rosinger AY, Bethancourt H, Swanson ZS, Nzunza R, Saunders J, Dhanasekar S, Kenney WL, Hu K, Douglass MJ, Ndiema E, 2021. Drinking water salinity is associated with hypertension and hyper-dilute urine among Daasanach pastoralists in Northern Kenya. Sci Total Environ. 770:144667. doi: 10.1016/j.scitotenv.2020.144667. PMC7969420 33515884

[R38] Sagawa T 2006. Wives’ domestic and political activities at home: the space of coffee drinking among the Daasanetch of Southwestern Ethiopia. Afr Stud Monogr. 27(2):63–86.

[R39] Sands JM, Layton HE. 2014. Advances in Understanding the Urine-Concentrating Mechanism. Annu Rev Physiol. 76(1):387–409. doi: 10.1146/annurev-physiol-021113-170350. 24245944

[R40] Sasaki JE, John D, Freedson PS. 2011. Validation and comparison of ActiGraph activity monitors. J Sci Med Sport. 14(5):411–416. doi: 10.1016/j.jsams.2011.04.003. 21616714

[R41] Sawka MN, Cheuvront SN, Carter R III. 2005. Human water needs. Nutr Rev. 63(6Pt 2):S30–S39. doi: 10.1111/j.1753-4887.2005.tb00152.x. 16028570

[R42] Shimamoto H, Komiya S. 2000. The turnover of body water as an indicator of health. J Physiol Anthropol Appl Human Sci. 19(5):207–212. doi: 10.2114/jpa.19.207. 11155349

[R43] Speakman JR, Yamada Y, Sagayama H, Berman ESF, Ainslie PN, Andersen LF, Anderson LJ, Arab L, Baddou I, Bedu-Addo K, 2021. A standard calculation methodology for human doubly labeled water studies. Cell Rep Med. 2(2):100203. doi: 10.1016/j.xcrm.2021.100203. PMC7897799 33665639

[R44] Swanson ZS. 2021. The effect of lifestyle change on health and early childhood growth in Daasanach Pastoralists Living in Northern Kenya [Internet]. [accessed 2023 Aug 14]. https://dukespace.lib.duke.edu/dspace/handle/10161/23028.

[R45] Swanson ZS, Pontzer H. 2020. Water turnover among human populations: effects of environment and lifestyle. Am J Hum Biol. 32(1):e23365. doi: 10.1002/ajhb.23365. 31782865

[R46] Tang R, Wang J-Y, Lo S-K, Hsieh L-L. 1999. Physical activity, water intake and risk of colorectal cancer in Taiwan: a hospital-based case-control study. Int J Cancer. 82(4):484–489. doi: 10.1002/(SICI)1097-0215(19990812)82:4<484::AID-IJC3>3.0.CO;2-A. 10404059

[R47] Thurber C, Dugas LR, Ocobock C, Carlson B, Speakman JR, Pontzer H. 2019. Extreme events reveal an alimentary limit on sustained maximal human energy expenditure. Sci Adv. 5(6):eaaw0341. doi: 10.1126/sciadv.aaw0341. PMC6551185 31183404

[R48] Tugjamba N, Walkerden G, Miller F. 2023. Adapting nomadic pastoralism to climate change. Clim Change. 176(4):28. doi: 10.1007/s10584-023-03509-0.

[R49] Urlacher SS, Snodgrass JJ, Dugas LR, Madimenos FC, Sugiyama LS, Liebert MA, Joyce CJ, Terán E, Pontzer H. 2021. Childhood daily energy expenditure does not decrease with market integration and is not related to adiposity in Amazonia. J Nutr. 151(3):695–704. doi: 10.1093/jn/nxaa361. 33454748

[R50] Yamada Y, Zhang X, Henderson MET, Sagayama H, Pontzer H, Watanabe D, Yoshida T, Kimura M, Ainslie PN, Andersen LF, 2022. Variation in human water turnover associated with environmental and lifestyle factors. Science. 378(6622):909–915. doi: 10.1126/science.abm8668. 36423296 PMC9764345

