# Supplementary material for: Total daily energy expenditure and elevated water turnover in a small-scale semi-nomadic pastoralist society from Northern Kenya
Source: Ann Hum Biol. Author manuscript; Available in PMC 2024 Nov 15. (PMC11567135; doi:10.1080/03014460.2024.2310724)
Supplement: supplemental information [file NIHMS2030572-supplement-supplemental_information.pdf]

Appendix - Supplementary Tables

Table S1. Full accelerometry data (wear days; moderate-vigorous, light and sedentary activity) for all accelerometry participants

Age	Sex	BM (kg)	TEE (kcal/day)	FFM (kg)	Fat %	water turnover (L/day)	mean daily steps	mean mins MVPA	mean mins light PA	mean mins sed.+ nonwear	wear days
31	F	57	2559	45.2	21.1	4.82	8483	52	155	1233	7
26	F	65	2794	45.9	29.0	13.56	3931	18	102	1320	7
30	F	46	2486	33.4	27.3	6.10	4409	17	100	1323	7
35	M	56	3494	52.0	7.3	11.11	10845	56	98	1286	7
45	M	49	3003	43.5	12.2	9.71	15776	33	345	1063	7
20	F	46	2419	36.2	20.7	6.15	5905	32	230	1169	4
31	F	45	3320	40.7	10.2	8.92	3059	13	97	1321	4
28	F	49	2044	39.3	19.6	7.04	4790	13	179	1238	5
25	M	54	3441	51.3	4.5	9.10	8907	66	112	1262	7
34	M	55	3143	48.9	10.8	9.39	7538	21	151	1268	7

Table S2. Summary data for comparative populations in water turnover analyses. Hadza data from Pontzer et al. (2015); Shuar data from Christopher et al. (2019).

Population	sex	n	mean age (yrs) (sd)	mean turnover (L/day) (sd)	mean lean mass (kg) (sd)	mean body fat % (sd)	annual temp (C)
Daasanach	F	19	28.9 (6.7)	7.26 (2.09)	36.9 (4.85)	24.9 (7.35)	20-37
Shuar	F	8	34 (14.9)	4.52 (0.34)	41.7 (3.54)	28.7 (6.17)	22-25
Hadza	F	18	41.1 (14.9)	3.74 (0.63)	34.2 (4.3)	20.8 (5.54)	25-30
Daasanach	M	16	36.4 (10.3)	7.43 (2.01)	45.6 (5.5)	14.0 (5.56)	20-37
Shuar	M	7	35 (15.3)	8.91 (2.23)	60.6 (7.07)	20.7 (6.67)	22-25
Hadza	M	15	35 (15.6)	4.43 (0.77)	43.6 (3.85)	12.2 (5.96)	25-20

Table S3. TEE models with community or population as interaction terms.

TEE across communities						
	lnTEE ~ (lnFFM + lnFM + sex) * community			lnTEE ~ (lnFFM + lnFM + age + sex) * community		
		community interaction			community interaction	
		El Bokoch	*Roto*		*El Bokoch*	*Roto*
	est. (se)	est. (se)	est. (se)	est. (se)	est. (se)	est. (se)
intercept	**4.32** (1.26)**	--	--	**3.93* (1.59)**	--	--
lnFFM (kg)	**1.06** (0.33)**	0.43 (0.50)	-0.26 (0.90)	**1.16** (0.39)**	0.47 (0.58)	-0.24 (0.95)
lnFM (kg)	-0.17 (0.13)	**0.65* (0.26)**	-0.07 (0.19)	-0.20 (0.14)	**0.68* (0.27)**	-0.03 (0.20)
age (yrs)	--	--	--	0.003 (0.006)	-0.008 (0.009)	-0.01 (0.01)
sex-M	-0.20 (0.13)	0.19 (0.20)	0.12 (0.26)	-0.26 (0.18)	0.26 (0.24)	0.19 (0.29)
community-El Bokoch	-3.18 (1.98)	--	--	-3.15 (2.23)	--	--
community-Roto	1.11 (3.4)	--	--	1.30 (3.59)	--	--
adj. R^2	0.68			0.66		

TEE across populations				
	lnTEE ~ (lnFFM + lnFM + age + sex) * population			
		population interaction terms		
		Hadza	*Shuar*	*Tsimane*
	est. (se)	est. (se)	est. (se)	est. (se)
intercept	**3.40*** (0.79)**	--	--	--
lnFFM (kg)	**1.32*** (0.20)**	-0.45 (0.31)	-0.25 (0.45)	**-0.55* (0.26)**
lnFM (kg)	**-0.14* (0.07)**	0.14 (0.08)	-0.04 (0.17)	**0.23* (0.09)**
age (yrs)	-0.002 (0.003)	0.001 (0.003)	0.008 (0.005)	0.0004 (0.004)
sex-M	**-0.19* (0.08)**	**0.31** (0.11)**	0.26 (0.19)	**0.26* (0.10)**
population-Hadza	1.06 (1.17)	--	--	--
population-Shuar	0.78 (1.60)	--	--	--
population-Tsimane’	1.39 (1.02)	--	--	--
adj. R^2	0.76			

Table S4. Water turnover models with community and population as interaction terms

water turnover across communities						
	turnover ~ (TEE + sex) * community			turnover ~ TEE * community		
		community interaction			community interaction	
		El Bokoch	*Roto*		*El Bokoch*	*Roto*
	est. (se)	est. (se)	est. (se)	est. (se)	est. (se)	est. (se)
intercept	-0.62 (2.16)			-0.25 (2.24)		
TEE (kcal/day)	**0.004*** (0.001)**	-0.002 (0.001)	**-0.003 (0.001)**	**0.003** (0.001)**	-0.002 (0.001)	-0.002 (0.001)
sex-M	**-2.05* (1.00)**	2.18 (1.41)	3.32 (1.67)	--	--	--
community-El Bokoch	4.12 (3.13)	--	--	3.71 (3.23)	--	--
community-Roto	6.56 (3.74)	--	--	4.78 (3.55)	--	--
adj. R^2	0.36			0.31		

water turnover across populations									
	turnover ~ TEE * population			turnover ~ (TEE + sex) *population			turnover ~ (FFM + FM + sex) * population		
		population interaction			population interaction			population interaction	
		Hadza	*Shuar*		*Hadza*	*Shuar*		*Hadza*	*Shuar*
	est. (se)	est. (se)	est. (se)	est. (se)	est. (se)	est. (se)	est. (se)	est. (se)	est. (se)
intercept	**3.08** (0.92)**	--	--	**3.02** (0.93)**	--	--	-1.47 (2.18)		
TEE (kcal/day)	**0.002*** (0.0004)**	-0.001 (0.001)	0.001 (0.001)	**0.002*** (0.0004)**	-0.001 (0.001)	0.0002 (0.001)	--		
FFM (kg)	--	--	--	--	--	--	**0.22*** (0.05)**	**-0.18* (0.08)**	-0.02 (3.65)
FM (kg)	--	--	--	--	--	--	0.05 (0.06)	-0.02 (0.09)	-0.02 (0.10)
sex-M	--	--	--	-0.44 (0.48)	0.70 (0.81)	1.55 (1.47)	**-1.28* (0.64)**	1.86 (1.05)	1.98 (1.65)
population-Hadza	-0.65 (1.33)			-0.34 (1.49)	--	--	3.23 (2.98)		
population-Shuar	**-4.97** (1.56)**			-3.71 (2.19)	--	--	-2.75 (3.65)		
adj. R^2	0.69			0.69			0.67		
